# Clinical Trial of the Virtual Integration Environment to Treat Phantom Limb Pain With Upper Extremity Amputation

**DOI:** 10.3389/fneur.2018.00770

**Published:** 2018-09-24

**Authors:** Briana N. Perry, Robert S. Armiger, Mikias Wolde, Kayla A. McFarland, Aimee L. Alphonso, Brett T. Monson, Paul F. Pasquina, Jack W. Tsao

**Affiliations:** ^1^Walter Reed National Military Medical Center, Bethesda, MD, United States; ^2^Applied Physics Laboratory, Johns Hopkins University, Laurel, MD, United States; ^3^Uniformed Services University of the Health Sciences, Bethesda, MD, United States; ^4^University of Tennessee Health Science Center, Memphis, TN, United States

**Keywords:** virtual reality therapy, upper extremity amputation, upper limb amputation, phantom limb pain, virtual integration environment, mirror therapy, neuropathic pain, surface electromyography (semg)

## Abstract

**Background:** Phantom limb pain (PLP) is commonly seen following upper extremity (UE) amputation. Use of both mirror therapy, which utilizes limb reflection in a mirror, and virtual reality therapy, which utilizes computer limb simulation, has been used to relieve PLP. We explored whether the Virtual Integration Environment (VIE), a virtual reality UE simulator, could be used as a therapy device to effectively treat PLP in individuals with UE amputation.

**Methods:** Participants with UE amputation and PLP were recruited at Walter Reed National Military Medical Center (WRNMMC) and instructed to follow the limb movements of a virtual avatar within the VIE system across a series of study sessions. At the end of each session, participants drove virtual avatar limb movements during a period of “free-play” utilizing surface electromyography recordings collected from their residual limbs. PLP and phantom limb sensations were assessed at baseline and following each session using the Visual Analog Scale (VAS) and Short Form McGill Pain Questionnaire (SF-MPQ), respectively. In addition, both measures were used to assess residual limb pain (RLP) at baseline and at each study session. In total, 14 male, active duty military personnel were recruited for the study.

**Results:** Of the 14 individuals recruited to the study, nine reported PLP at the time of screening. Eight of these individuals completed the study, while one withdrew after three sessions and thus is not included in the final analysis. Five of these eight individuals noted RLP at baseline. Participants completed an average of 18, 30-min sessions with the VIE leading to a significant reduction in PLP in seven of the eight (88%) affected limbs and a reduction in RLP in four of the five (80%) affected limbs. The same user reported an increase in PLP and RLP across sessions. All participants who denied RLP at baseline (*n* = 3) continued to deny RLP at each study session.

**Conclusions:** Success with the VIE system confirms its application as a non-invasive and low-cost therapy option for PLP and phantom limb symptoms for individuals with upper limb loss.

## Introduction

By the year 2050, it is estimated that almost 3.6 million persons will be living with amputations within the United States (1). As of March 2018, military conflicts in Iraq and Afghanistan have resulted in 1,719 United States military service members sustaining major limb loss, with 297 (17.3%) losing an upper limb (J.C. Shero, personal communication, April 4, 2018). Persons who have sustained a major limb amputation suffer from a unique set of challenges. Following limb loss, almost everyone experiences phantom limb sensations, which include the perception of itching, pressure, or temperature changes in the phantom limb, as well as an awareness of its orientation in space (2). Furthermore, reports estimate that 85% of all persons with amputation experience painful sensations, or phantom limb pain (PLP), either immediately following amputation or within days to weeks post-operation (3). For many, both phantom sensations and PLP are bothersome and even disabling, interfering with the ability to live independently and further emphasizing the need for successful treatment interventions.

Numerous pharmacological interventions for the treatment of PLP have been explored (4). These interventions remain, however, largely ineffective long-term (5). Of the non-pharmacological and non-invasive therapy options, mirror therapy has proven successful in treating PLP in the majority of cases (6–25). Mirror therapy involves placing a mirror along the midline of a person with a unilateral amputation to generate a reflection of his or her intact limb such that both limbs appear present. This provides the individual with a visual representation of the phantom limb moving in space. In a study by Chan et al. 18 individuals with unilateral UE amputation and PLP received either mirror, covered-mirror, or mental visualization therapy for 15 min a day for 4 weeks. Within the mirror group, all 6 (100%) participants experienced PLP relief. Comparatively, only one participant (17%) in the covered-mirror group and two participants (33%) in the mental visualization group had pain relief, with multiple individuals even reporting a worsening of their pain (10). A subsequent study by Tung et al. investigated the role of mirror treatment for PLP in individuals with bilateral lower extremity amputations finding that the direct visual observation of another person's limb movements also effectively decreases pain (11).

Despite the frequency of phantom sensations and PLP after limb amputation, the pathophysiology remains largely unknown (9). It has been hypothesized that it is the visual feedback component of mirror therapy that disrupts the phantom pain experience, which is supported by studies demonstrating pain relief with mirror therapy as opposed to covered-mirror therapy or mental visualization practices alone (10, 11, 24, 25). The results from both mirror and observational therapy studies lead us to postulate that motor imagery created in a virtual environment may also be effective in treating PLP. To date, a few case studies have successfully used virtual visual feedback to reduce PLP, often noting a pain reduction in persons who were resistant to previously attempted therapies (26–35). In a study by Mercier et al. eight individuals with UE amputation and PLP observed and followed along with the movements of a virtual limb twice a week for 8 weeks. By the end of the study, five of the participants (63%) reported at least a 30% reduction in PLP, supporting the use of virtual reality therapy to treat PLP (27).

Herein we describe the initial clinical testing of the Virtual Integration Environment (VIE) platform among users who sustained upper extremity (UE) amputation. This platform was designed by the Johns Hopkins University Applied Physical Lab (JHU/APL) and is a virtual reality stimulator. Users of the VIE platform can both passively follow along with and actively command the muscle movements of a virtual avatar using surface electromyography (EMG) signals captured from their residual limbs (36–38). In this study, we sought to evaluate the use of the VIE platform as a PLP therapy for individuals with UE loss.

## Materials and methods

### Participants

**Figure 1:**
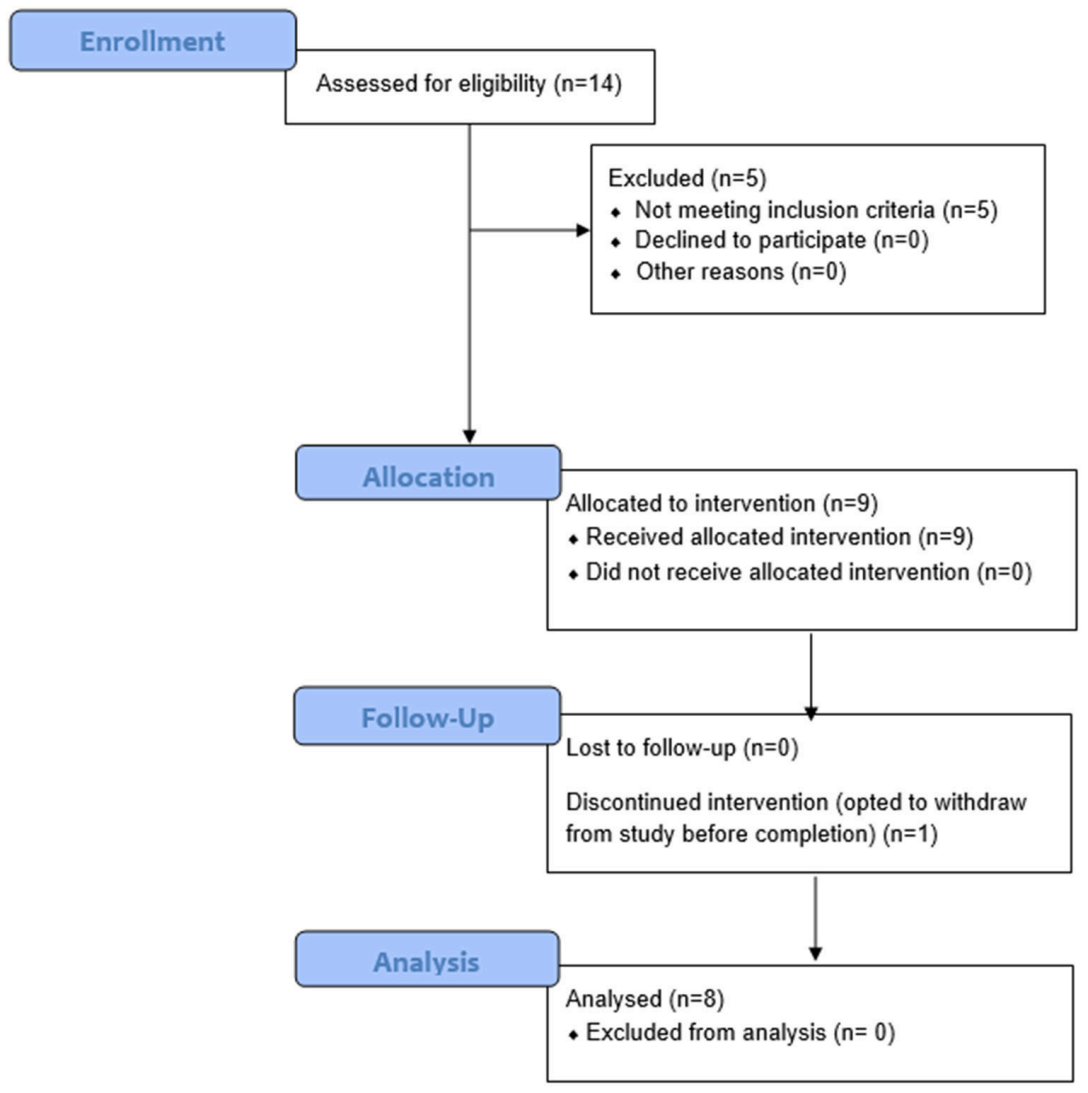
Consort Flow Diagram. Fourteen individuals were recruited and consented for this study. Of these 14 persons, nine had phantom limb pain (PLP) at baseline assessment as defined by the Visual Analog Scale (VAS). Of these nine persons, eight completed the study and were thus included in the final analysis. Participant 6 withdrew from the study after three sessions due to scheduling conflicts. Of the remaining eight participants, five reported residual limb pain (RLP) at baseline, as defined by their VAS scores.

For the clinical trial “Virtual Integration Environment in Decreasing Phantom Limb Pain,” identifier number NCT01462461 (ClinicalTrials.gov), volunteers were recruited at Walter Reed National Military Medical Center (WRNMMC) in Bethesda, MD, within 18 months of sustaining an UE amputation. Data collection occurred from 10/18/2011 through 5/10/2014. The Institutional Review Board (IRB) at WRNMMC gave approval for the study, and written informed consent was obtained from all participants. In addition to the presence of an UE amputation, inclusion criteria consisted of a normal neurological examination (except for amputation), the presence of three weekly PLP episodes at the time of enrollment, and no prior history of vertebral disk disease/condition, sciatica, or radiculopathy. Exclusion criteria included the presence of traumatic brain injury, known uncontrolled systemic disease, significant DSM-IV Axis I or II diagnosis (39) in the 6 months prior to enrollment, and a score lower than a 42/50 on the Test of Memory Malingering (TOMM). In total, 14 individuals were recruited for and consented to this study at WRNMMC in Bethesda, MD, between October 2011 and May 2014 (Figure 1).

### System components

**Figure 2:**
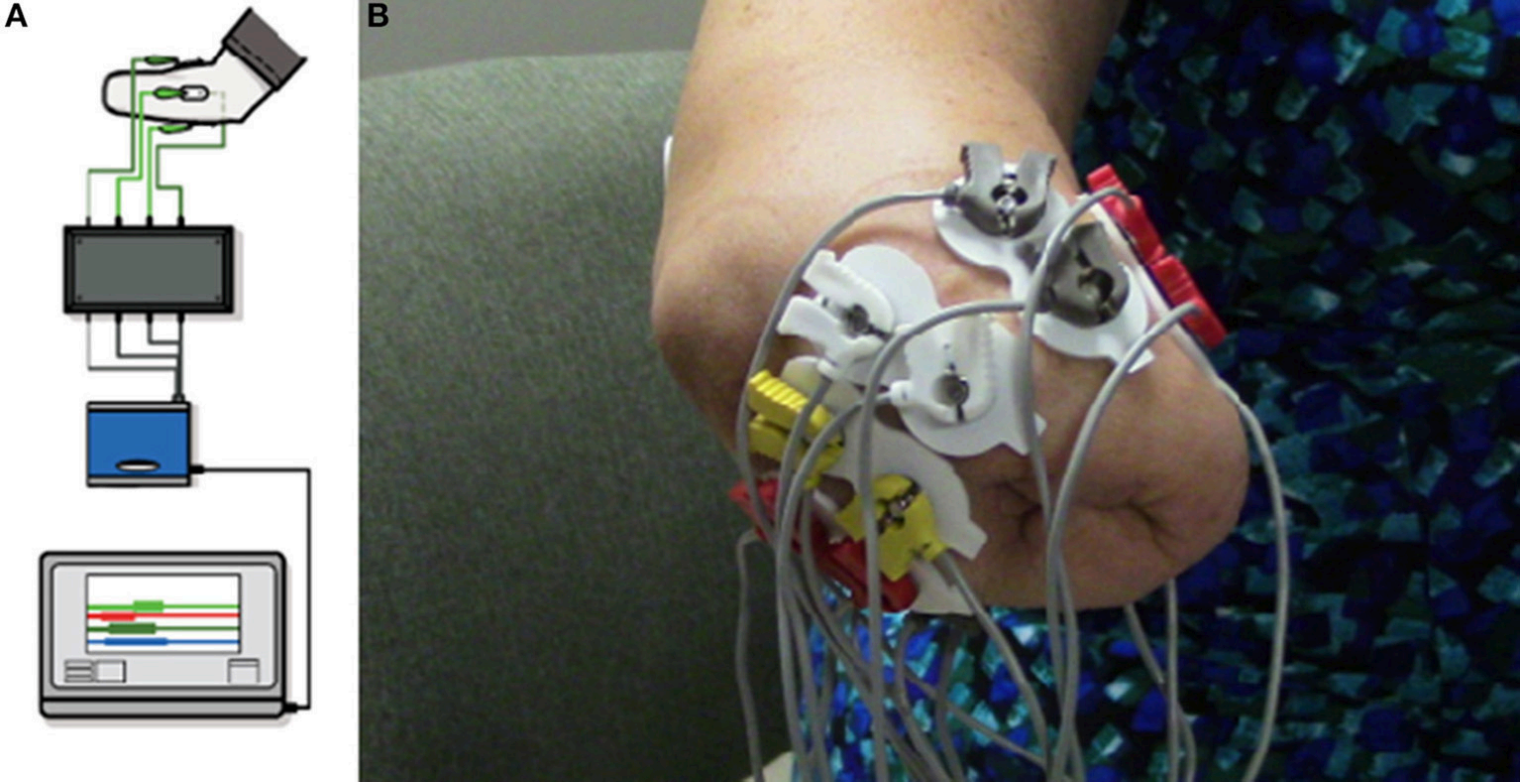
Virtual Integration Environment (VIE) Set-Up and Electrode Configuration. Using MiniVIE open source code, created in affiliation with the John Hopkins University Applied Physics Laboratory and available at https://bitbucket.org/rarmiger/minivie, myoelectric signal processing was used to execute pattern recognition training and virtual avatar limb control. **(A)** illustrates the various VIE components: live motor data collection, signal filtering and processing, pattern classification and machine learning modules, and user assessments of classifier performance (36, 40). **(B)** demonstrates the placement of eight pairs of bipolar surface electromyography electrodes circumferentially around the user's residual limb. One ground electrode is positioned either below the elbow or below the shoulder depending on residual limb length.

The VIE system runs on a laptop computer using both an operator screen and a visualization screen. The VIE has five core sub-systems: inputs, signal analysis, controls, plant, and presentation. The input modules are compatible with a wide variety of sources, including cortical inputs, surface EMG signals, or intramuscular EMG signals. For this study, the input was surface EMG signals (36–38). Eight bipolar electrode pairs were placed circumferentially around the residual limb, as well as one ground electrode either below the elbow (in the case of individuals with trans-radial amputation) or below the shoulder (in the case of individuals with trans-humeral amputation). EMG signals were then digitized via an electrically isolated data acquisition system (Figure 2). Signal analysis algorithms within the VIE performed EMG signal filtering, signal feature extraction, and classification using machine learning-based pattern recognition software. The control and plant sub-systems translated user-intended motions into individual joint commands resulting in motion of the entire virtual arm. The system output presentation displayed a rendered 3-D arm within the VIE environment, observed by the user on the visualization screen. The rendered environment was based on the Musculo-Skeletal Modeling Software allowing for stereoscopic display (41). In addition to the virtual environment, the VIE synchronizes with a physical prosthetic limb system, allowing seamless transition from virtual to physical limb control (40). The most recent implementation of the VIE used for this study is the open-source MiniVIE code project, part of The Open Prosthetics Project (http://openprosthetics.org/). The MiniVIE code project reflects the concepts and workflow of the JHU/APL VIE platform, but is a separate and lightweight MATLAB-based implementation. The VIE was specifically designed to synchronize with the Modular Prosthetic Limb (MPL), an advanced myoelectric prosthetic arm designed by JHU/APL for DARPA Revolutionizing Prosthetics 2009 (42–44), but has the potential to synchronize with a variety of myoelectric prostheses.

### VIE procedure

All participants were screened, enrolled, and consented by a member of the WRNMMC research team before beginning to participate in the study. The study aimed to have participants complete virtual therapy across 20, 30-min sessions over the course of 1 to 2 months. The initial session included a brief introduction to the VIE system.

#### Pain surveys

At each session, participants completed a Phantom Limb Pain Survey comprised of 10-cm Visual Analog Scales (VAS), which were used to quantify the PLP, and the Short-Form McGill Pain Questionnaire (SF-MPQ), which was used to characterize the PLP. Additional VAS and SF-MPQ questions assessed any RLP that was present. The VAS is a simple and minimally intrusive measure of pain, which has been widely used in clinical and research settings and found to be valid and internally consistent (45). The SF-MPQ is a brief questionnaire that is frequently employed to assess the occurrence, severity, and symptoms of pain (46).

#### Motion control

**Figure 3:**
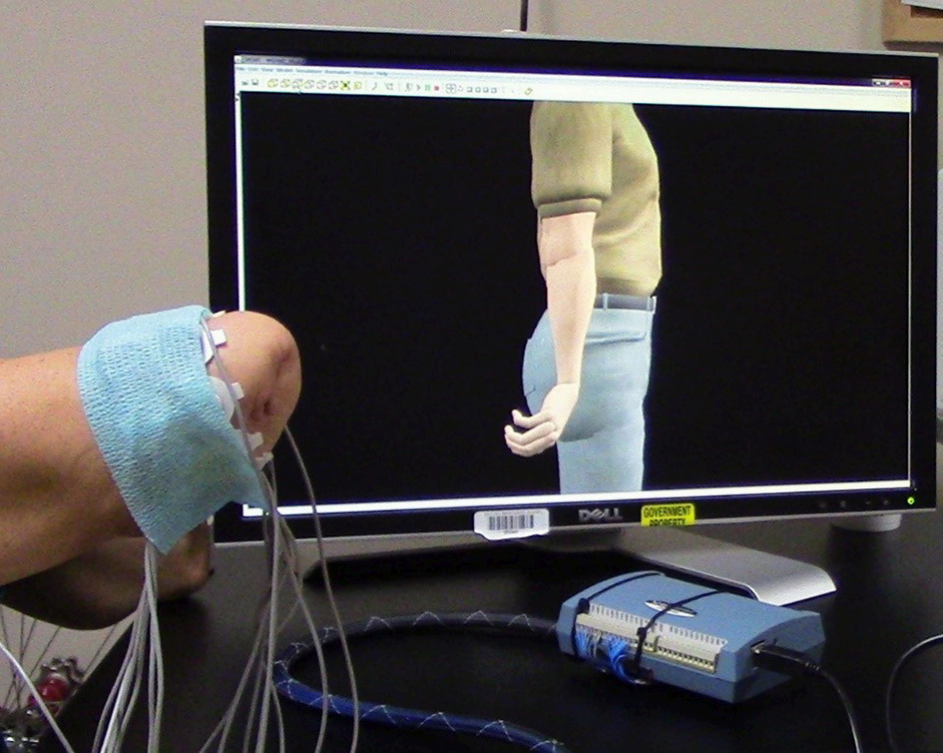
Training with the Virtual Integration Environment (VIE). A study participant is seen during a VIE training session where he observes the virtual avatar limb moving through a set of physiological motions while mentally visualizing his phantom limb completing those same movements. Surface electromyography (EMG) electrodes are placed circumferentially around his residual limb to ensure that he is following along with the program and creating unique muscle contraction patterns for each motions. These same EMG recordings are later used during a period of “free-play” where he drives the virtual avatar limb through the previously trained motion classes using signal capture from his residual limb.

Training with the VIE consisted of 20, 30-min visualization sessions in which the participant observed a virtual avatar's limb moving automatically through physiological ranges of motion (Figure 3). Participants were instructed to mentally follow the movements with their phantom limb. Surface EMG data was simultaneously recorded from the residual limbs of these participants using eight bipolar electrodes placed circumferentially around the participants' residual limbs. The cued motion of the passive virtual limb was used to label the surface EMG recordings. The movements conducted were wrist flexion and extension, wrist pronation and supination, and hand opening and closing to form a fist. At the start of each session, the motion types were presented in a set sequence. At the end of each session, the computer generated a randomized order of motion type presentation. Each motion was executed by the virtual limb in multiple, 2-min intervals. The collection of EMG signals was used to ensure that participants were actively engaged throughout the therapy session. Moreover, we sought to see whether system users were creating consistent muscle patterns with each prompted movement.

After completion of the 30-min visualization session, participants were given the option of engaging in a period of “free-play” within the VIE system where they could utilize the surface EMG signal capture from their residual limb to drive virtual avatar limb movements. These movements were the same as those used during the visualization session (i.e., wrist flexion and extension, wrist pronation and supination, and hand opening and closing to form a fist).

### VIE assessment

#### Pain survey assessment

To complete the VAS portion of the Phantom Limb Pain Survey, participants were asked to mark three 10-cm lines at places corresponding to the severity of their “current PLP,” “average PLP” (over the last 24 h), and “worst PLP” (over the last 24 h) on a scale of “no pain” to the “worst pain that someone could ever experience.” Additionally, participants were asked to mark three VAS lines, with similar scales, at places corresponding to the severity of their “current RLP,” “average RLP,” and “worst RLP.” The VAS values were measured as the distance in cm from the location on the line corresponding to “no pain” (i.e., 0 cm) to the point on the line marked by the participant, with a maximum value of 10 cm.

To complete the SF-MPQ portion of the survey, participants were asked to rate the intensity of 15 pain descriptors as severe, moderate, mild, or none. These intensities corresponded to pain scores of three, two, one, or zero, respectively, and were summated to generate the daily total SF-MPQ score for each participant. This score both highly correlates to and is sensitive to the effect of pain treatments (46).

Statistical analysis of both the VAS and SF-MPQ results was completed using a univariable linear mixed effect regression model. This statistical method accounts for clustering of data points within subjects, inconsistent testing intervals, and missing data. To account for clustering within subjects, a random intercept was used. All analyses were conducted using R version 3.4.2 with statistical significance defined as *p* < 0.05 (47). All statistical tests were two-tailed.

## Results

### Participants

Of the 14 participants recruited to this study, nine reported PLP at screening. Of these nine individuals, eight completed the VIE study. The ninth participant withdrew after three sessions due to scheduling conflicts and is therefore not considered in the final analysis. Of the eight participants who completed the study, five additionally reported RLP at baseline. All participants were male, active duty military personnel between 20 and 30 years of age (Table 1). They sustained their amputations within 6–18 months prior to their enrollment in the study. Seven of the individuals had unilateral UE amputation, while one had bilateral UE amputation. Due to other military commitments, each participant was not always able to complete all 20 sessions. On average, the eight participants completed 17.9 ± 4.0 sessions over 79.9 ± 46.3 days.

**Table 1:** Participant Demographics.

**Participant ID**	**Amputation site, side**	**Months since amputation**	**RLP**
1	ED, Left	14	No
2	TH, Right	9	Yes
3	TH, Right	18	Yes
4	TR, Left	18	Yes
7	TR, Left	13	Yes
8	WD, Right	6	No
13	WD, Right	6	No
14	TR, Left	10	Yes

*Participant ID, amputation details (site, side, and months since amputation), and residual limb pain (RLP) status are provided for the eight individuals who completed this study. Participant 06 withdrew after three sessions due to scheduling conflicts and is, therefore, not reflected. Participants 05 and 09-12 reported no phantom limb pain (PLP) at baseline and thus were excluded from the study. The following abbreviations describe the amputation site: ED, elbow disarticulation; TH, trans-humeral; TR, trans-radial; WD, wrist disarticulation*.

### VIE results

#### VAS results

**Figure 4:**
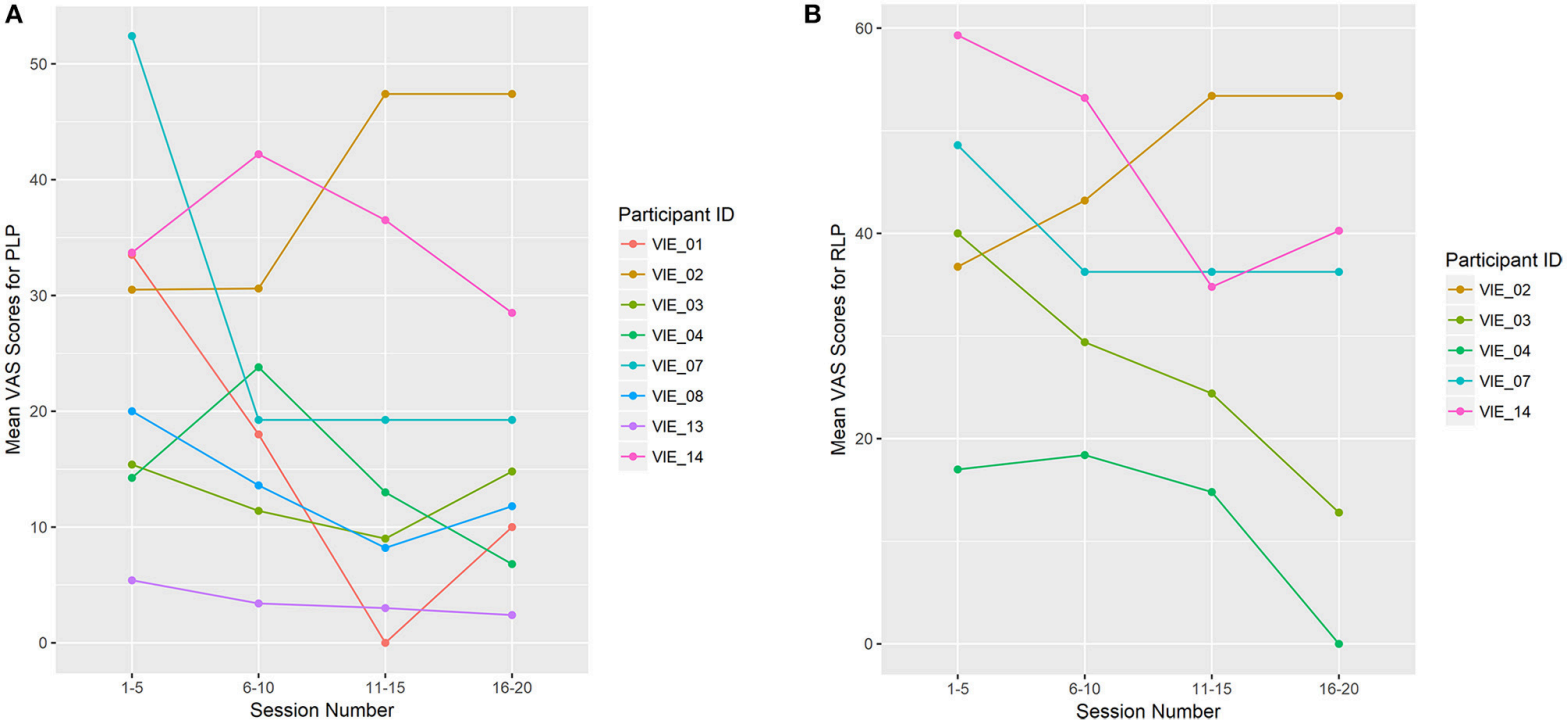
Limb Pain Scores **(A)** shows the mean Visual Analog Scale (VAS) scores for the worst phantom limb pain (PLP) experienced during the previous 24 h. PLP decreased across study sessions in seven of the eight phantom limbs (88%), which was significant (β = −0.474, *p* = 0.015). **(B)** shows the mean VAS scores for the worst residual limb pain (RLP) experienced during the previous 24 h. RLP decreased across study sessions in four of the five (80%) residual limbs. The remaining four participants denied RLP at baseline, as well as throughout the study. For display purposes, study sessions are divided into four groups: 1–5, 6–10, 11–15, and 16–20. Each data point represents the mean VAS score for that user throughout the session grouping.

Overall, PLP decreased in seven of the eight (88%) phantom limbs across study sessions. The “worst PLP” VAS scores improved significantly across the study (β = −0.474, *p* = 0.015; Figure 4), as did the “current PLP” scores (β = −0.248, *p* = 0.042). While the “average PLP” scores improved across the study, the change was not significant (β = −0.295, *p* = 0.078). By the completion of the VIE study, RLP had decreased in four of the five individuals (80%) who had reported it present at baseline. The same individual who reported an increase in PLP from baseline to completion of the study was also the individual who reported an increase in RLP across sessions (i.e. participant 2).

#### SF-MPQ results

**Figure 5:**
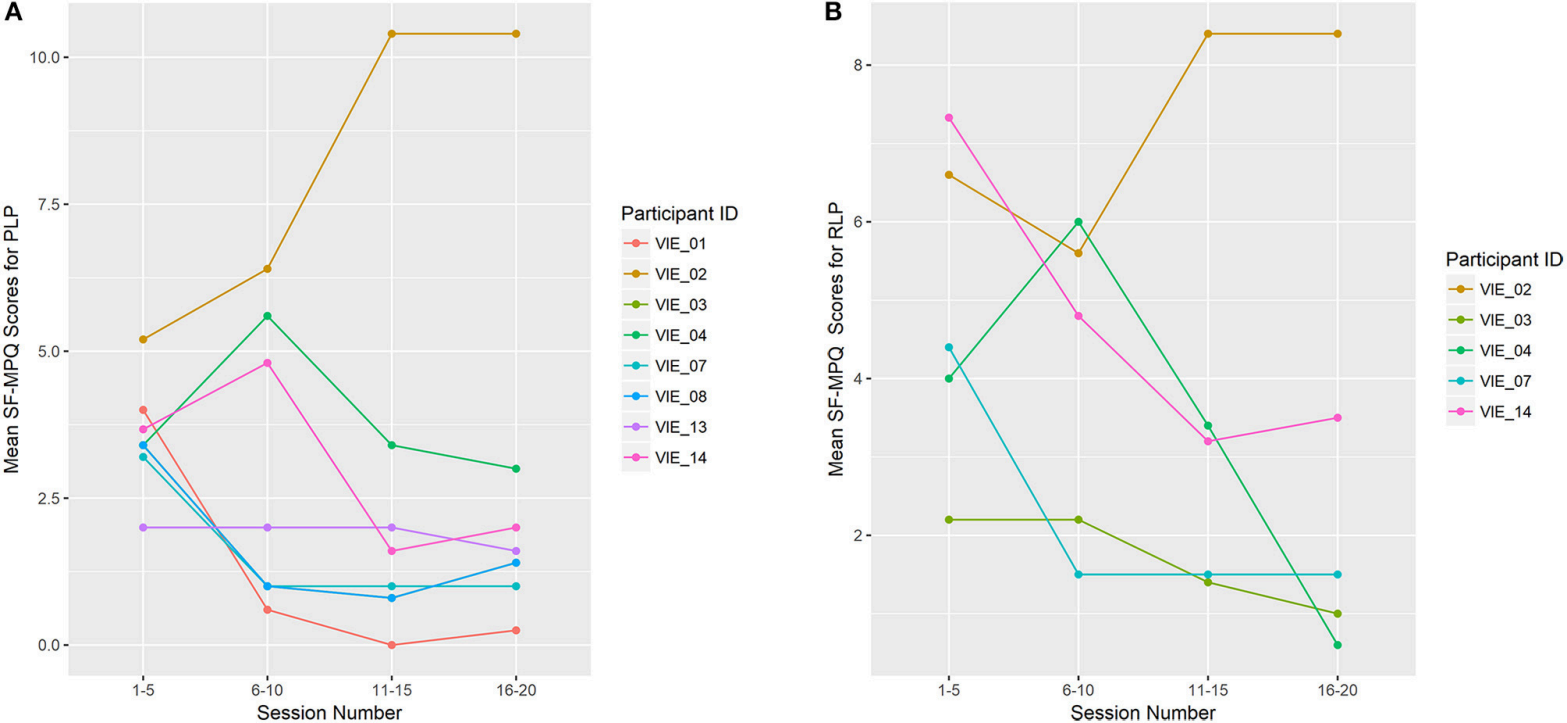
Limb Pain Symptoms **(A)** shows the mean total Short-Form McGill Pain Questionnaire (SF-MPQ) scores for participants who reported phantom limb pain (PLP; *n* = 8). **(B)** shows the mean total SF-MPQ scores for participants who reported residual limb pain (RLP; *n* = 5). Study sessions are condensed into four time-points: 1–5, 6–10, 11–15, and 16–20. Each data point represents the mean total SF-MPQ score across that session grouping. VIE treatment lead to a decline in phantom limb symptom burden for 7 of the 8 participants (88%), which was a significant change (β = −0.096, *p* = 0.003), and a decline in the residual limb symptom burden for 4 of the 5 participants (80%).

Overall, the total SF-MPQ scores of seven of the eight (88%) participants decreased across study sessions, which was a significant improvement (β = −0.096, *p* = 0.003; Figure 5). Similarly, the SF-MPQ scores of four of the five (80%) participants with RLP decreased across study sessions. The PLP descriptors most frequently reported were “sharp,” “stabbing,” and “throbbing,” and the RLP descriptors most frequently reported were “aching,” “tender,” and “throbbing.”

#### EMG results

EMG signal capture collected in real time from surface electrodes on the residual limbs of the participants confirmed that users were actively engaged throughout the VIE study. Moreover, grouping of the EMG signals based on similarity and labeling with the motion class prompts demonstrated that unique motion patterns were being generated for each prompted motion. The surface EMG data collected was utilized each session to allow for participants to engage in a period of “free-play” where they actively drove the movements of the virtual avatar's limb.

## Discussion

Seven of the eight (88%) participants who completed this study had a significant reduction in PLP and phantom limb symptoms across sessions, as defined by the VAS and SF-MPQ scores, respectively (23, 34). Furthermore, of the five participants who reported RLP, four (80%) noted a decrease in RLP and residual limb symptoms across sessions. These results suggest that the VIE is a viable PLP and RLP therapy option for the majority of individuals with UE amputation. No individual who denied RLP at baseline developed RLP while training with the VIE. Interestingly, it was the same participant who reported an increase in PLP and in RLP across study sessions. The exact reason that this individual was a non-responder is unknown, but could be explained by a global lack of attention to the training program or an inability to isolate movements with his phantom limb. Importantly, the individuals who did demonstrate themselves to be VIE responders noted relief in all aspects of their limb pain (i.e., phantom and residual).

The promising pain reduction seen with the VIE platform lends support to our hypothesis that virtual reality therapy can be used to effectively treat PLP with individuals with UE amputation. The idea of using visual feedback to treat PLP has primarily been explored using mirror therapy studies, however multiple case studies have begun to investigate the use of virtual visual feedback for pain relief (6–35). In addition to the successful use of the VIE platform by participants with unilateral UE amputation, this study included the successful treatment of PLP in one participant with bilateral UE amputation. This is particularly important as mirror therapy relies on the presence of an intact limb on either the user or a colleague to generate a reflected intact limb (8–23). Comparatively, we have demonstrated that the VIE allows for an individual with bilateral UE amputation to undergo pain relief therapy alone, without requiring the assistance of a colleague.

Limitations of this study include the small sample size, the differences in baseline PLP between participants, the differences in amputation location along the upper limb, and the differences in total user exposure to the VIE therapy. As the participants were active duty veterans they were at times completing physical and occupational therapy while in this study. These therapies were difficult to monitor and could not be limited for the sake of the study as they were integral to their overall recovery. Without a control group we are unable to compare changes in PLP in participants receiving the intervention vs. those who were not. It is possible that a placebo effect is responsible for some degree of the pain relief reported. The participants here sustained their amputations within two years of study enrollment (specifically 6–18 months), and it is difficult to know how much their pain would have improved over time alone. Future studies should aim to have a larger sample size overall and per amputation site, and to analyze participants according to their time since amputation, as well as compared to a control group.

In this study, we demonstrated that a virtual system can be used to significantly reduce PLP in individuals with UE amputation. Participants demonstrated the ability to move their phantom limb in concert with a virtual avatar and elicit surface EMG signals unique to those motions. These findings suggest that using a virtual system, such as the VIE, to provide a visual feedback component to motor imagery therapy represents a viable treatment option for PLP and RLP.

## Ethics statement

This study was carried out in accordance with the recommendations of Department of Research Programs, Walter Reed National Military Medical Center (WRNMMC) with written informed consent from all subjects. All subjects gave written informed consent in accordance with the Declaration of Helsinki. The protocol was approved by the WRNMMC Institutional Review Board.

## Author contributions

BP led the study administration, data analysis, and manuscript production. RA led the technical support and contributed to study design and data analysis. MW assisted with study administration and data analysis. KM assisted with study administration and manuscript writing. AA assisted with study administration and manuscript writing. BM assisted with study design and study administration. PP oversaw study design and study administration. JT oversaw data analysis and manuscript production.

### Conflict of interest statement

The authors declare that the research was conducted in the absence of any commercial or financial relationships that could be construed as a potential conflict of interest.
